# Integrated transcriptomic analysis identifies tsRNA–mRNA regulatory axis in asthma pathogenesis^☆^

**DOI:** 10.1016/j.waojou.2026.101389

**Published:** 2026-04-28

**Authors:** Ying Zhou, Hang Lin, Dongmei Zhou, Xinhui Gong, Ximeng Ma, Lei Shi, Yubao Cui

**Affiliations:** a Department of Pediatrics Laboratory, The Affiliated Children's Hospital of Jiangnan University, Wuxi 214023, China; b Department of Allergy, The Affiliated Hospital of Qingdao University, Qingdao 266003, China; c Clinical Research Center, The Affiliated Wuxi People's Hospital of Nanjing Medical University, Wuxi 214023, China; d Department of Clinical Laboratory, Shuguang Hospital Affiliated to Shanghai University of Chinese Traditional Medicine, Shanghai 201203, China

**Keywords:** Asthma, Bronchial epithelial cells, tsRNA, mRNA, Biomarkers

## Abstract

**Background:**

Asthma is a complex respiratory disorder with incompletely understood molecular mechanisms. tRNA-derived small RNAs (tsRNAs), a newly discovered class of non-coding RNAs, have been implicated in various diseases, but their role in asthma pathogenesis remains largely unexplored.

**Methods:**

We performed an integrated analysis of tsRNA and mRNA profiles in bronchial epithelial cells from severe asthma patients (GSE85215, GSE85214) and healthy controls. Differentially expressed tsRNAs were identified using MINTmap and DESeq2 (*P* < 0.05, |FC| > 1.5), and their target mRNAs were predicted via miRanda and TargetScan. Validation was conducted in an independent cohort (GSE201955). Functional interactions were assessed using a tsRNA–mRNA regulatory network, and key findings were experimentally confirmed in house dust mite (HDM)-stimulated BEAS-2B cells via qRT-PCR and dual-luciferase reporter assays.

**Results:**

We identified 58 dysregulated tsRNAs and 961 differentially expressed mRNAs in asthma. A regulatory network revealed 343 negatively correlated tsRNA–mRNA pairs, with 36 hub mRNAs validated in GSE201955. Three candidate tsRNAs (tRF-18-SX73IE03, tRF-22-BZBZOS4Y1, tRF-22-MI7O3B1N4) showed consistent dysregulation in HDM-exposed cells. Mechanistically, tRF-21-Z4NY2V7KE directly suppressed MYBL1 expression, as confirmed by luciferase assays.

**Conclusions:**

Our study provides the first comprehensive tsRNA–mRNA regulatory network in asthma, uncovering novel post-transcriptional mechanisms in bronchial epithelial cells. The identified tsRNAs and their targets may be potential biomarkers; moreover, they warrant further investigation as therapeutic targets for asthma.

## Introduction

Asthma is a chronic inflammatory disorder marked by airway inflammation, bronchial hyperresponsiveness, and reversible airflow obstruction, with global prevalence varying significantly across regions, ranging from 3.44% in Asia and 3.67% in Africa to 4.90% in South America, 5.69% in Europe, 8.29% in North America, and 8.33% in Oceania.^1^ It is triggered by environmental factors such as allergens (eg, pollen, dust mites, pet dander), respiratory infections, tobacco smoke, and air pollutants. Genetic predisposition also significantly contributes to the development of asthma, as certain genetic variants can increase an individual's susceptibility to the condition.^2^^,^^3^ This complex interplay of environmental and genetic factors results in diverse asthma manifestations, including variations in symptom triggers, disease severity, and treatment responses, posing challenges in effective diagnosis and management.^4^

Non-coding RNAs (ncRNAs) have emerged as key regulators of many biological processes, including inflammation and immune responses. They can be categorized into 3 groups based on their length: long ncRNAs (>200 nt), such as ribosomal RNA (rRNA) and long non-coding RNA (lncRNA); medium-length ncRNAs (40–200 nt), like transfer RNA (tRNA) fragments, small nucleolar RNA (snoRNA), and small nuclear RNA (snRNA); and short ncRNAs (<40 nt), including microRNA (miRNA), piwi-interacting RNA (piRNA), and tRNA-derived small RNAs (tsRNAs). Research has highlighted the roles of lncRNAs, circRNAs, and miRNAs in asthma pathogenesis, demonstrating their involvement in immune modulation and airway inflammation.^5^^,^^6^ Among these, macrophages and T helper cells are crucial in asthma development. Macrophages influence airway inflammation, immune regulation, and airway function through ncRNA-mediated polarization.^5^^,^^7^, ^8^, ^9^, ^10^ tsRNAs, a novel subclass of small non-coding RNAs, regulate diverse cellular processes including gene expression control, epigenetic modulation, and intercellular communication, critically influencing cell fate determination and metabolic homeostasis.^11^, ^12^, ^13^ While tsRNA dysregulation has been implicated in various diseases, their expression and function in asthma have yet to be investigated.

Bronchial epithelial cells (BECs), key players in the airway defense system, protect the airways from harmful substances and facilitate the removal of particulates through mucociliary clearance.^14^, ^15^, ^16^ These cells also participate in immune responses by releasing lipid mediators, cytokines, and growth factors involved in asthma pathogenesis.^17^, ^18^, ^19^ Despite the known dysregulation of various ncRNAs in asthma, the role of tsRNAs in BECs during disease pathogenesis remains largely unexplored. Therefore, this is a study aimed to investigate the tsRNA expression profile in BECs from severe asthma patients and to elucidate the potential tsRNA-mRNA regulatory networks involved in disease development.

## Materials and methods

### Datasets and samples

We searched the GEO database for “asthma” and “Bronchial Epithelial Cells” and successfully downloaded the GSE85216 and GSE201955 datasets. GSE85216 includes RNA-Seq data from GSE85214 and miRNA-Seq data from GSE85215, both derived from bronchial epithelial cells of patients with severe asthma and healthy controls.

**Supplementary Fig. 1:**
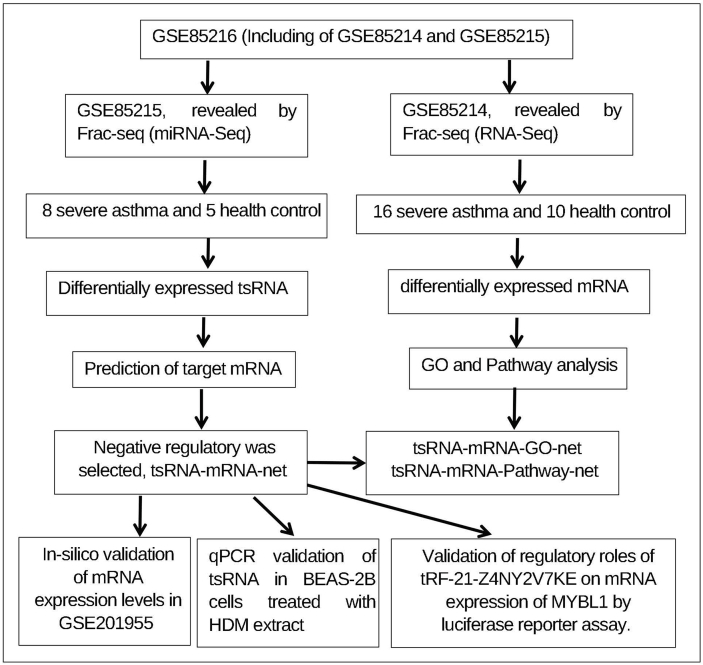
The flow chart shows the overall design of the study

Specifically, the mRNA expression profile was analyzed using GSE85214 (16 severe asthma patients vs. 10 healthy controls). For tsRNA analysis, the original FASTQ files from GSE85215 (8 severe asthma patients vs. 5 healthy controls) were processed. Validation of mRNA expression was performed using GSE201955, an independent cohort comprising 79 asthma patients (including individuals with clinical features associated with severe asthma endotypes, as characterized by Magnaye et al)^20^ and 39 healthy controls (Supplementary Fig. 1).

### Differential expression tsRNA analysis

The original fastq data were downloaded from the GSE85215 with 8 severe asthma and 5 health control individuals. The MINTmap (v2.0) was used to screen and quantify tsRNA,^21^ and then differentially expressed tsRNAs were identified between asthma and control using the DESeq2 package in R, with a filtering threshold of P < 0.05 and |FC| > 1.5.

### Construction of tsRNA-mRNA regulatory network

To construct the tsRNA-mRNA regulatory network, we first analyzed RNA-seq data from bronchial epithelial cells (GSE85214: 16 severe asthma vs. 10 healthy controls) using DESeq2 (v1.38.3) with |log_2_FC| > 1.5 and adjusted *P* < 0.05 (Benjamini-Hochberg correction) to identify differentially expressed mRNAs (DEmRNAs), while tsRNA targets were predicted by integrating miRanda (v3.3a; ΔG ≤ −20 kcal/mol, seed complementarity) and TargetScan (v7.0; context++ score >50) to ensure high-confidence interactions, followed by filtering for anti-correlated pairs (Pearson's r < −0.5, *P* < 0.05) between tsRNA and mRNA expression levels, with the resulting network visualized in Cytoscape (v3.9.1) and functionally annotated using DAVID (v6.8) for GO/KEGG enrichment (*P* < 0.05, Fisher's exact test) to elucidate asthma-related pathways.

### *In-silico* validation of mRNA expression levels

To validate the mRNA expression levels in the GSE85214 dataset, a total of 36 mRNA with a *P* value lower than 0.05 were selected, which was then detected in the GSE201955 dataset.

### qRT-PCR validation of tsRNA in BEAS-2B cells stimulated with mite extract

qRT-PCR was performed to validate the expression of differentially expressed tsRNAs in BEAS-2B cells (American Type Culture Collection, Rockville, MD) treated with house dust mite (HDM) extract. The cells were cultured in RPMI-1640 medium supplemented with 10% (v/v) heat-inactivated FBS (HyClone, Logan, UT, USA). BEAS-2B cells were seeded in 6-well plates at a density of 150,000 cells per well, and after 24 h, 40 μg/mL of HDM extract (*Dermatophagoides pteronyssinus*, Greer Laboratories, Lenoir, NC, USA) was added. After another 24 h, total RNA was extracted using TRI Reagent (Sigma: T9424, Sigma-Aldrich, Saint Louis, MO, USA). RNA pre-treatment and cDNA synthesis were then performed using the rtStar™ tRF&tiRNA Pretreatment Kit (Cat# AS-FS-005) and rtStar™ First-Strand cDNA Synthesis Kit (Cat# AS-FS-003). RNA was first deacetylated by mixing it with the deacetylation reaction buffer and incubating at 37 °C for 40 min, followed by the addition of Deacylation Stop Buffer and a 5-min room temperature incubation. Next, 3′-cP was removed and 5′-P was added, and the mixture was incubated at 37 °C for 40 min, followed by a 5-min incubation at 70 °C to terminate the reaction. The RNA was extracted again, and demethylation was performed by mixing the Demethylation Reaction Buffer with Demethylase and incubating at 37 °C for 2 h. The reaction was then terminated with the addition of the termination solution, followed by RNA extraction. For 3′ adaptor ligation, RNA was mixed with the 3′ adaptor, incubated at 70 °C for 2 min, and then cooled on ice before adding the reaction mixture, which was incubated at 25 °C for 1 h. Reverse transcription primer hybridization was carried out by adding the reverse transcription primer and incubating at specified temperatures. Next, 5′ adaptor ligation was performed by mixing the 5′ adaptor with the appropriate reagents and incubating at 25 °C for 1 h. Finally, reverse transcription was performed by adding the adaptor ligated RNA and reverse transcription reagents, incubating at 50 °C for 1 h, and cooling the reaction for subsequent PCR amplification.

Real-time PCR was conducted using the Applied Biosystems QuantStudio™ 5 Real-Time PCR System with 2X PCR master mix (Arraystar) and Primer 5.0 software. To prepare the standard curve, cDNA templates for both target and housekeeping genes were selected. PCR reactions were set up using 2 × Master Mix, 10 μM forward and reverse primers, cDNA, and water to a final volume of 10 μl. The PCR conditions were 95 °C for 10 min, followed by 40 cycles of 95 °C for 10 s and 60 °C for 60 s for fluorescence collection. PCR products were analyzed on a 2% agarose gel with a 100 bp DNA Ladder to confirm the amplification specificity. The PCR products were serially diluted (1 × 10^−1^ to 1 × 10^−9^) to create the standard curve. For real-time PCR, cDNA samples were mixed with 2 × Master Mix, primers, and water, then briefly centrifuged. The reaction mixture was added to a 384-well PCR plate, followed by 2 μl of cDNA, sealed with Sealing Film, briefly centrifuged, and kept on ice before PCR. The PCR program included 95 °C for 10 min, followed by 40 cycles of 95 °C for 10 s and 60 °C for 60 s with fluorescence collection. After amplification, a melt curve was generated by heating from 60 °C to 99 °C. The system automatically calculated the relative gene expression by normalizing the target gene concentrations to housekeeping gene levels based on the standard curve. The qPCR primer sequences are provided in Supplementary Table 1.

Prior to the formal tsRNA validation experiments, the responsiveness of BEAS-2B cells to HDM stimulation under the current conditions was confirmed in our laboratory through preliminary assays (eg, observing typical morphological changes and increased expression of inflammatory markers, data not shown). Furthermore, this model has been widely adopted and validated in numerous studies.^22^^,^^23^ The consistency of our preliminary findings with this extensive body of literature confirms the suitability of this well-established model for the current study.

### Dual-luciferase reporter gene assay

The 3ʹ-untranslated regions (UTRs) of MYBL1 (containing a tRF-21-Z4NY2V7KE binding site) were synthetized and cloned into pmirGLO vector (Promega) to construct wild-type plasmids. The 3ʹ-UTRs of MYBL1 was mutated from “TCTATGTATGGAAAGCACA” to “CTCGCACACAGGGGATGTG”, which was synthesized and cloned into pmirGLO vector (Promega) to construct mutation-type plasmids. The Mimics of tRF-21-Z4NY2V7KE was synthesized, too.

As we previously described in detail,^24^ the HEK 293T cells (the Cell Bank of the Chinese Academy of Sciences, Shanghai, China) were cultured on a 96-well culture plate 24 h before transfection. The culture medium was Dulbecco's Modified Eagle Medium (DMEM) + 10% Fetal bovine serum (FBS). The plasmids were subjected to transfection at 60%–70% confluency. After 48 h, a Dual-Luciferase Reporter Gene Assay Kit (11402ES60, Yeasen Biotechnology, Shanghai, China) was used to assay luciferase activity to verify the effect of tRF-21-Z4NY2V7KE on MYBL1.

### Statistical analysis

Numerical data are presented as mean ± standard deviation (SD). Differential expression analysis of tsRNAs and mRNAs between asthmatic patients and healthy controls (GSE85214, GSE85215) was performed using DESeq2 (v1.38.3) with thresholds of |log_2_FC| > 1.5 and adjusted *P* < 0.05 (Benjamini-Hochberg correction for multiple testing). For validation in the independent cohort (GSE201955), Student's t-test was applied to compare expression levels of 36 hub mRNAs, with significance set at *P* < 0.05. Differential expression of tsRNAs in HDM-stimulated BEAS-2B cells was assessed via Student's t-test (version 22.0 SPSS, Chicago, USA), and *P* < 0.05 was deemed statistically significant. The Pearson correlation analysis for tsRNA-mRNA pairs (|cor| > 0.5, *P* < 0.05) was adjusted for multiple comparisons using the false discovery rate (FDR) method. All tests were two-tailed, and variance homogeneity was confirmed via Levene's test.

## Results

### Construction of tsRNA-mRNA regulatory network

**Fig. 1:**
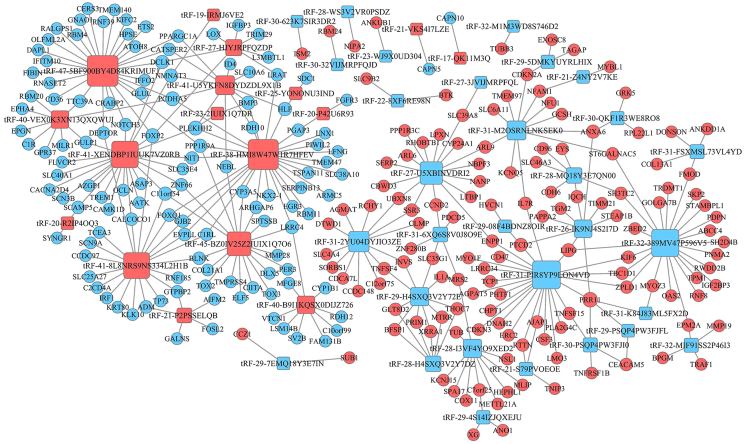
Regulatory network of tsRNA-mRNA interactions in asthma pathogenesis. The network illustrates 343 negatively correlated tsRNA-mRNA pairs identified in bronchial epithelial cells, with rectangles representing tsRNAs and circles representing mRNAs. Red indicates upregulation, blue indicates downregulation, and node size reflects regulatory strength (larger size = stronger correlation). Key asthma-related pathways (eg, inflammatory response, airway remodeling) are enriched among the hub genes (eg, *MYBL1*, *CEACAM5*), validated in an independent cohort (GSE201955). This network highlights novel post-transcriptional regulatory mechanisms, with experimentally confirmed suppression of *MYBL1* by tRF-21-Z4NY2V7KE, suggesting potential therapeutic targets for asthma

24 severe asthmatics and 15 health controls in the GSE85216 dataset (including GSE85214 and GSE85215)^25^ were enrolled in the construction of tsRNA-mRNA regulatory network. We initially analyzed the differential expression of tsRNAs between 8 severe asthmatic patients and 5 health control individuals in the GSE85215 dataset, followed by an analysis of mRNA expression levels between 16 severe asthma and 10 health control in the GSE85214 dataset. The construction and validation process of the tsRNA-mRNA regulatory network is illustrated in Supplementary Fig. 1. In total, 58 differentially expressed tsRNAs (Table 1) and 961 differentially expressed mRNAs were observed in bronchial epithelial cells. According to the detection results, we constructed the negative regulatory networks with a cutoff |cor|>0.5 using the Cytoscape software (for details, see the Supplementary Methods in the Online Repository). Consequently, the interactions between tsRNAs and mRNAs were identified, and subsequent statistical analysis revealed 343 pairs of tsRNA-mRNA negative regulatory relationships, encompassing 47 tsRNAs and 254 mRNAs (Fig. 1 and Supplementary Table 2).

**Table 1:** Differentially expressed tsRNAs between severe asthma and health control individuals

tsRNA MINTbase_ID	sequence	log2FoldChange	style	*P* value
tRF-29-PSQP4PW3FJFL	GCCCGGCTAGCTCAGTCGGTAGAGCATGA	−2.237056712	Down	0.000246017
tRF-30-PSQP4PW3FJI0	GCCCGGCTAGCTCAGTCGGTAGAGCATGAG	−2.319316189	Down	0.000421713
tRF-23-WJ9X0UD304	TCGGCTGTTAACCGAAAGGTTGG	−3.811301691	Down	0.001466312
tRF-23-BZBZOS4YV	AACTTAACTTGACCGCTCTGACC	2.026751696	Up	0.001638739
tRF-22-MI7O3B1N4	CGGCTGTTAACCGAAAGGTTGG	−3.056880169	Down	0.001866166
tRF-38-HMI8W47W1R7HFEV	ATATCATTGGTCGTGGTTGTAGTCCGTGCGAGAATACC	3.623880688	Up	0.002438001
tRF-27-HJYJRPFQZDP	ATAGCTTAGCGGTAGAGCATTTGACTG	3.635608675	Up	0.003127083
tRF-20-R2IP4OQ3	GGGGAATTAGCTCAAGCGGT	2.479625852	Up	0.004078512
tRF-29-PS5P4PW3FJF2	GCCCGGATAGCTCAGTCGGTAGAGCATCA	−2.769672856	Down	0.004147541
tRF-22-BZBZOS4Y1	AACTTAACTTGACCGCTCTGAC	2.487907694	Up	0.004457195
tRF-31-M2OSRNLNKSEK0	CGGGAGACCGGGGTTCGATTCCCCGACGGGG	−2.123681434	Down	0.005010545
tRF-28-WS3V2VR0PSDZ	TCTCGCCTGCCACGCGGGAGGCCCGGGT	−2.0629518	Down	0.005199811
tRF-30-623K7SIR3DR2	GGAGACCGGGGTTCGATTCCCCGACGGGGA	−3.051878437	Down	0.006135064
tRF-18-SX73IE03	GTCTAGTGGTATGATTCT	3.458903754	Up	0.006226498
tRF-22-79M89P9NI	GTTTCCGTGGTGTAGTGGTTAT	−3.041543093	Down	0.007618769
tRF-31-2YU04DYJIO3ZE	CACTGTAAAGCTAACTTAGCATTAACCTTTT	−2.160897127	Down	0.007706691
tRF-40-VEX0K3XN13QXQWUI	TAGATTGAAGCCAGTTGATTAGGGTGCTTAGCTGTTAACT	2.822629124	Up	0.008886318
tRF-17-QK11M3Q	GCGGGAGGCCCGGGTTT	2.509487698	Up	0.010021056
tRF-21-P2PSSELQB	GCAGAGCCCGGTAATCGCATA	3.157411323	Up	0.010522779
tRF-41-8L8NRS9NS334L2H1B	TCATATCATTGGTCGTGGTTGTAGTCCGTGCGAGAATACCA	2.363698707	Up	0.010698882
tRF-20-P42U6R93	GCATGCACGAGGCCCTGGGT	3.499451928	Up	0.010931776
tRF-41-U5YKFN8DYDZDL9X1B	TACACTTAGGAGATTTCAACTTAACTTGACCGCTCTGACCA	2.952835454	Up	0.011588804
tRF-28-I3VF4YO9XED2	ATGGTTAGCACTCTGGACTCTGAATCCA	−2.184612733	Down	0.012404392
tRF-32-MIF91SS2P46I3	CGGCTAGCTCAGTCGGTAGAGCATGGGACTCT	−2.017163523	Down	0.013074589
tRF-23-2IUIX1Q7DR	CAACTTAACTTGACCGCTCTGAC	3.307885712	Up	0.013116275
tRF-22-8XF6RE98 N	TCCTAAGCCAGGGATTGTGGGT	−2.56829377	Down	0.014052447
tRF-27-W3FJI0E7UM5	TCGGTAGAGCATGAGACTCTTAATCTC	−2.393448977	Down	0.015314791
tRF-29-4S14IZJQXEJU	CTCCGAGGTGATTTTCATATTGAATTGCA	−2.570921148	Down	0.016629727
tRF-30-QKF1R3WE8RO8	GCGGGAGACCGGGGTTCGATTCCCCGACGG	−2.161384893	Down	0.017478753
tRF-32-389MV47P596V5	CCTGGTGGTCTAGTGGTTAGGATTCGGCGCTC	−2.122703863	Down	0.017799856
tRF-31-PIR8YP9LON4VD	GCACTGGTGGTTCAGTGGTAGAATTCTCGCC	−2.081762507	Down	0.018212393
tRF-31-6XQ6S8V0J8O9E	GGCTAGCTCAGTCGGTAGAGCATGGGACTCT	−2.344294423	Down	0.018392052
tRF-29-5DMKYUYRLHIX	GAGACCGGGGTTCGATTCCCCGACGGGGA	−2.014030483	Down	0.01840148
tRF-22-WD8SS46D2	TCGACTCCCGGTATGGGAACCA	−2.068479477	Down	0.019257342
tRF-21-S79PVOEOE	GTCCTTGTAGTATAAACTAAT	−2.064471302	Down	0.020264274
tRF-31-FSXMSL73VL4YD	AGCCGTGATCGTATAGTGGTTAGTACTCTGC	−2.353283973	Down	0.02048367
tRF-19-18VUY9IQ	AGTGGTATGATTCTCGCTT	2.413774246	Up	0.021500326
tRF-21-Z4NY2V7KE	TTGTGCTTTGCACGCGTGGGT	−2.457420207	Down	0.021642073
tRF-19-IRMJ6VE2	ATTCCCGGGCGGCGCACCA	2.148014499	Up	0.022174462
tRF-29-08F4BDNZ8OIR	ACCGGAGATGAAAACCTTTTTCCAAGGAC	−2.408449035	Down	0.023887304
tRF-18-43RQ590K	CTAGTGGTATGATTCTCG	2.02458771	Up	0.024214957
tRF-25-YONONU3IND	TTCAACTTAACTTGACCGCTCTGAC	2.558763424	Up	0.02478296
tRF-31-K84J83ML5FX2D	CCCGGCTAGCTCAGTCGGTAGAGCATGAGAC	−2.057152992	Down	0.025374427
tRF-30-32VIJMRPFQJD	CCGGATAGCTCAGTCGGTAGAGCATCAGAC	−2.179503679	Down	0.026146605
tRF-40-B9I1KQSX0DIJZ726	AACTCATGCCCCCATGTCTAACAACATGGCTTTCTCACCA	2.233331282	Up	0.02631471
tRF-28-H4SXQ3V2Y7DZ	ATATGGTCTAGCGGTTAGGATTCCTGGT	−2.16710546	Down	0.026412491
tRF-45-BZ0IV25Z2IUIX1Q7O6	AACTTACACTTAGGAGATTTCAACTTAACTTGACCGCTCTGACCA	2.682135899	Up	0.030493817
tRF-26-IK9NJ4S2I7D	ATGGGTGGTTCAGTGGTAGAATTCTC	−2.13392983	Down	0.031145948
tRF-28-MQ18Y3E7QN00	CGTATAGTGGTTAGTACTCTGCGTTGTG	−2.010816348	Down	0.035847172
tRF-21-VKS4I7LZE	TAGGGGTATGATTCTCGCTTT	2.570463757	Up	0.037271046
tRF-17-8Z1MQ8Q	TCCTTAGGTCGCTGGTT	−2.022022763	Down	0.039077026
tRF-27-U5XBINVDRI2	TACACTGAAAATGTTTAGACGGGCTCA	−2.319668237	Down	0.0395488
tRF-32-M1M3WD8S746D2	CGGCCCGGGTTCGACTCCCGGTGTGGGAACCA	−2.303735617	Down	0.040428234
tRF-27-3JVIJMRPFQL	CCGGCTAGCTCAGTCGGTAGAGCATGA	−2.128434561	Down	0.040476564
tRF-29-H4SXQ3V2Y72E	ATATGGTCTAGCGGTTAGGATTCCTGGTT	−2.245440821	Down	0.040570509
tRF-41-XENDBP1IUUK7VZ0RB	TGAATCTGACAACAGAGGCTTACGACCCCTTATTTACCCCA	2.107607172	Up	0.040735393
tRF-29-7EMQ18Y3E7IN	GTGATCGTATAGTGGTTAGTACTCTGCGT	−2.115583704	Down	0.048812002
tRF-47-5BF900BY4D84KRIMUF1	GAGAAAGCTCACAAGAACTGCTAACTCATGCCCCCATGTCTAACAAC	2.846945402	Up	0.049174852

**Supplementary Fig. 2:**
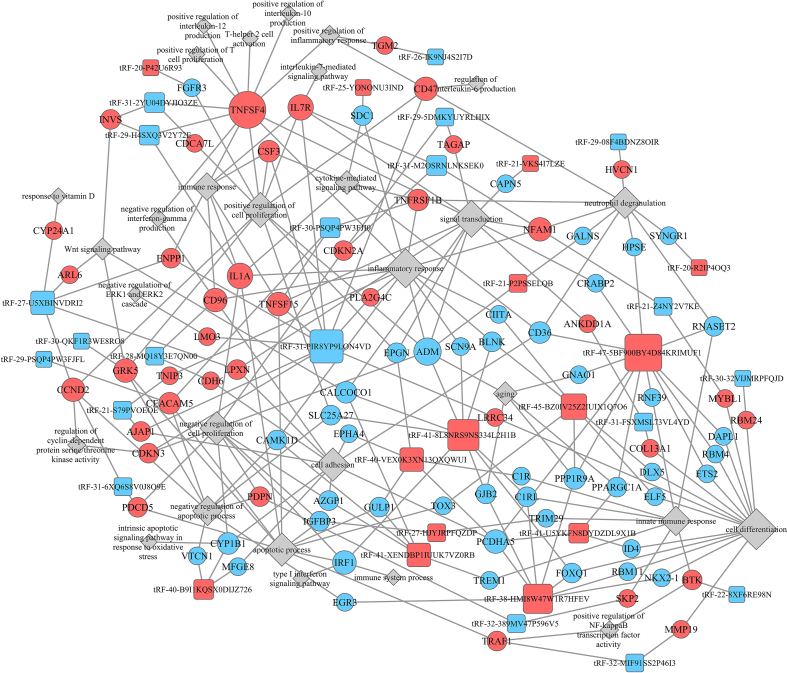
GO enrichment of tsRNA-mRNA network. **Note:** Rectangle represents tsRNA, rotundity represents mRNA, diamond represents GO, red represents up-regulation, blue represents down-regulation, and gray represents GO. The regulation abilities increase with the size of the graphs

**Supplementary Fig. 3:**
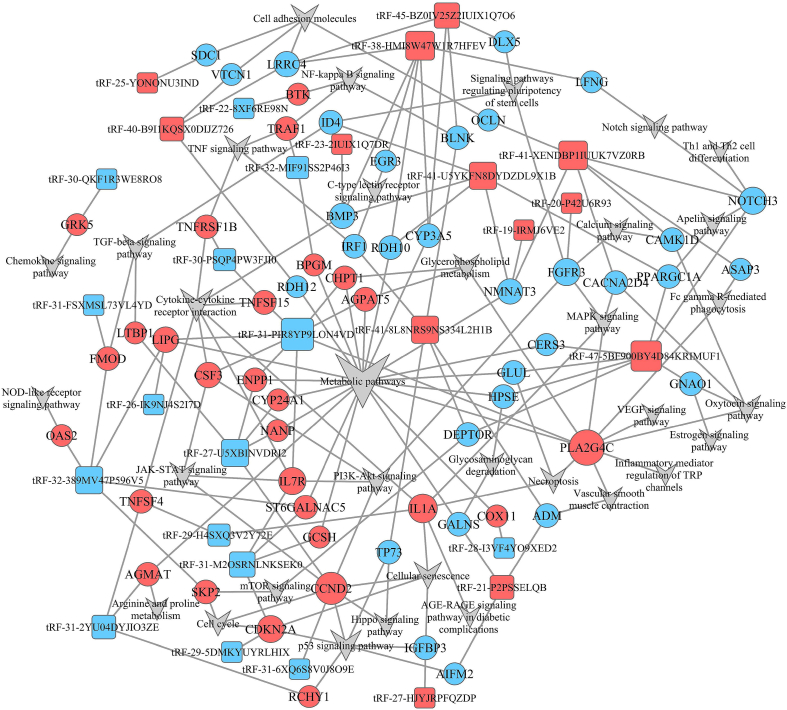
KEGG-Pathway of tsRNA-mRNA network. **Note:** Rectangle represents tsRNA, rotundity represents mRNA, V-shaped represents pathway, red represents up-regulation, blue represents down-regulation, and gray represents pathway. The regulation abilities increase with the size of the graphs

To further investigate the potential functional implications of the tsRNA, we performed functional enrichment analyses for the 961 mRNAs and 343 tsRNA-mRNA pairs by respectively using GO enrichment analyses and KEGG-Pathway analyses. As shown in Supplementary Fig. 2, the GO enrichment highlighted the comprehensive regulatory functions of these tsRNAs on mRNAs. Furthermore, the KEGG-Pathway analyses detailed the pathway of tsRNAs in regulating mRNAs (Supplementary Fig. 3). GO/KEGG analyses of this network implicated these tsRNA-mRNA axes in key asthma pathways, including IL-17 signaling and airway remodeling.

### *In-silico* validation of differentially expressed mRNAs

**Fig. 2:**
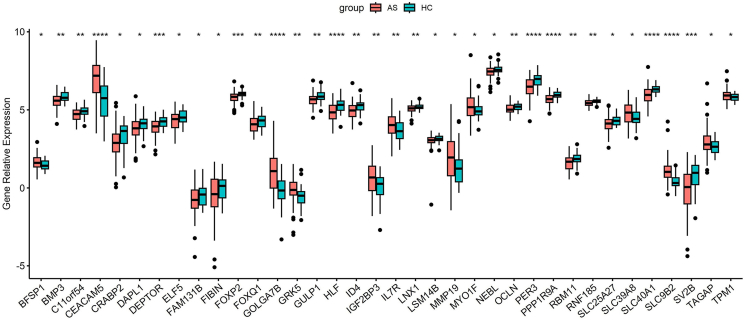
Validation of 36 asthma-associated mRNAs (GSE85214) in GSE201955 dataset. **Note:** Differential expression (*P* < 0.05) confirmed in 79 AS vs 39 HC. AS, asthma; HC, healthy control

Based on the constructed tsRNA-mRNA negative regulatory relationships, we selected 36 hub mRNAs with a *P* value lower than 0.05 from the discovery cohort (GSE85214, severe asthma patients vs. healthy controls). We then examined the expression levels of these candidates in an independent validation cohort (GSE201955), which comprises 79 asthmatics (including individuals with clinical features associated with severe asthma endotypes) and 39 healthy controls. Notably, all 36 selected mRNAs exhibited significant differential expression in this validation cohort (all *P* < 0.05, Fig. 2), confirming the reproducibility of our findings across different asthma populations.

### qRT-PCR validation of tsRNAs in BEAS-2B cells stimulated with *Dermatophagoides pteronyssinus* extract

**Fig. 3:**
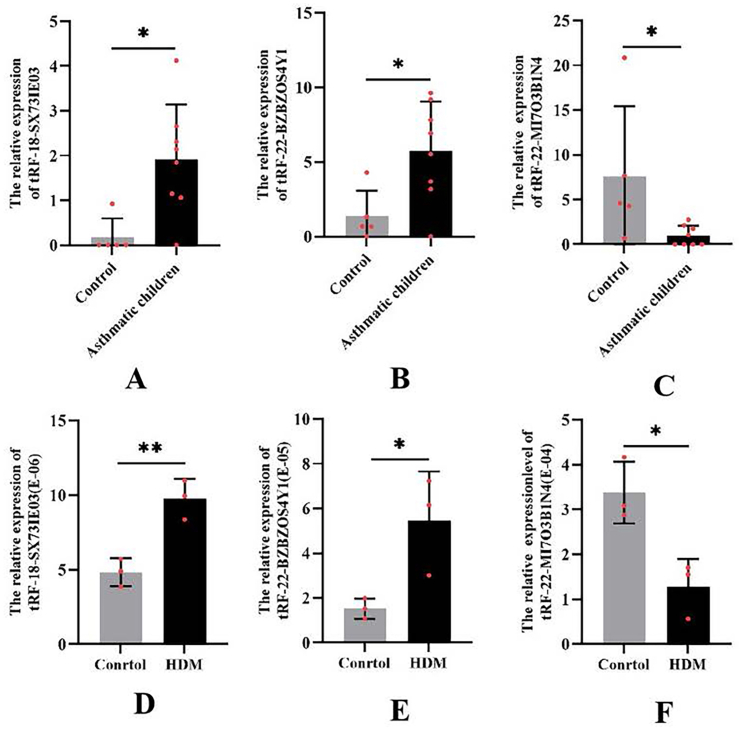
Validation of 3 tsRNAs in HDM-stimulated BEAS-2B cells. (**A-C**) Expression levels of tRF-18-SX73IE03, tRF-22-BZBZOS4Y1, and tRF-22-MI7O3B1N4 from the GSE85215 dataset. (D–F) qRT-PCR validation of these tsRNAs in BEAS-2B cells treated with house dust mite extract (40 mg/mL). Data represent mean ± SEM from 3 independent experiments. HDM, house dust mite

To verify the accuracy of tsRNA expression levels presented in the above transcriptome sequencing results, we used the human bronchial epithelial cells being exposed to house dust mite extract as the cell model of asthma. We cultured BEAS-2B cells with 40 μg/mL of house dust mite extracts, and then amplified tsRNAs for validation via quantitative real-time PCR (qRT-PCR). The detection results confirmed significant differences in the expression levels of tRF-18-SX73IE03 (*P* < 0.01), tRF-22-BZBZOS4Y1 (*P* < 0.05), and tRF-22-MI7O3B1N4 (*P* < 0.05) compared to controls (Fig. 3).

### Verification of tRF-21-Z4NY2V7KE regulating mRNA expression of MYBL1 by luciferase reporter assay

**Fig. 4:**
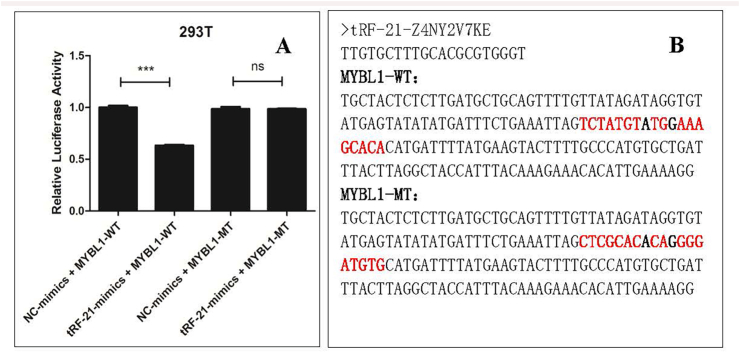
Validation of tRF-21-Z4NY2V7KE targeting MYBL1 through luciferase reporter assay. (A) Relative dual-luciferase activity demonstrating tRF-21-Z4NY2V7KE's regulatory effect on MYBL1 3′UTR (∗∗∗*P* < 0.001 *vs.* control). (B) Sequence alignment of tRF-21-Z4NY2V7KE with wild-type (WT) and mutant (MT) MYBL1 3′UTR binding sites. Data represent mean ± SD from 3 independent experiments

Using a dual-luciferase reporter gene system and site-directed mutagenesis, we investigated the potential regulatory effect of tRF-21-Z4NY2V7KE on the MYBL1 gene. The results showed that tRF-21-Z4NY2V7KE down-regulated luciferase expression in the MYBL1 context (*P* < 0.01, Fig. 4). The altered base sequences suggest that tRF-21-Z4NY2V7KE modulates MYBL1 mRNA expression through its affinity for 3ʹ URT binding sites, indicating a specific post-transcriptional regulatory mechanism.

## Discussion

In this study, we investigated the role of tsRNAs in asthma pathogenesis by conducting an integrated transcriptomic analysis of bronchial epithelial cells from severe asthma patients. Our analysis revealed a previously uncharacterized tsRNA-mRNA regulatory network, identifying 58 differentially expressed tsRNAs and 961 differentially expressed mRNAs. Notably, we constructed 343 negatively correlated regulatory pairs and validated the dysregulation of key tsRNAs (tRF-18-SX73IE03, tRF-22-BZBZOS4Y1, and tRF-22-MI7O3B1N4) in an HDM-stimulated cellular model. Furthermore, mechanistic studies confirmed that tRF-21-Z4NY2V7KE directly suppresses MYBL1 expression via 3′UTR binding. These findings position tsRNAs as critical post-transcriptional regulators in asthma and provide a foundational framework for understanding their role in airway inflammation.

BECs play a crucial role in the airway defense system, preventing harmful substances from entering the airways and facilitating the removal of particulates through mucociliary clearance and other mechanical barriers.^24^ Beyond their protective functions, BECs actively participate in immune responses by producing and releasing biologically active compounds such as lipid mediators, growth factors, endothelin, and a variety of cytokines/chemokines. These mediators are integral to the pathogenesis of airway disorders, including asthma.^18^ Previous studies have demonstrated that gene expression profiles in BECs differ significantly between healthy individuals and those with severe asthma. For instance, transcriptomic analysis has identified over 1200 differentially expressed genes between central and peripheral airways in asthma patients.^26^ Further investigation combining transcriptomic data from severe asthma patients and healthy controls revealed a total of 1703 differentially expressed genes and 71 microRNAs, offering insights into novel inflammatory and structural mechanisms contributing to asthma.^27^ While these studies have established BECs as a valuable model for asthma research and identified numerous protein-coding transcripts involved in disease pathogenesis, the regulatory roles of non-coding RNAs, particularly transfer RNA-derived small RNAs (tsRNAs), remain largely unexplored in asthma.

To address this knowledge gap and build upon existing transcriptomic findings, we conducted an integrated analysis of mRNA and tsRNA profiles in bronchial epithelial cells using the GSE85216 dataset (GSE85214 and GSE85215) created by Rocio T. Martinez-Nunez et al (2018).^25^ Our study revealed 961 differentially expressed mRNAs and 58 tsRNAs in severe asthma patients compared to healthy controls. Significantly, we established the first comprehensive tsRNA-mRNA interactome for asthma, identifying 343 negatively correlated regulatory pairs (Pearson's r < −0.5, *P* < 0.05). Among these, 36 hub mRNAs demonstrated consistent dysregulation in an independent validation cohort (GSE201955; 79 asthma vs 39 controls). GO and pathway analyses further confirmed these tsRNA-mRNA interactions participate in asthma-related biological processes. The reproducibility of these findings across cohorts and their convergence with known asthma mechanisms not only validates prior work on BEC dysregulation but importantly expands our understanding of ncRNA regulation in asthma pathogenesis. These robust molecular signatures highlight promising diagnostic biomarkers and provide new insights into asthma's complex regulatory networks.

Building upon recent breakthroughs in tsRNA diagnostics, such as the identification of tRF-Ala-AGC-2-M4 as a specific serum biomarker for lupus nephritis,^28^ our study demonstrates parallel diagnostic potential for asthma through the discovery of 3 clinically relevant tsRNAs. Using an established HDM-stimulated bronchial epithelial cell model, we identified and validated tRF-18-SX73IE03, tRF-22-BZBZOS4Y1, and tRF-22-MI7O3B1N4 through rigorous qRT-PCR confirmation, mirroring the validation approach that successfully established tRF-Ala-AGC-2-M4 as a lupus nephritis biomarker.^28^ Importantly, our findings show these asthma-associated tsRNAs share key characteristics with their lupus nephritis counterparts: (1) disease-specific expression patterns (consistent with GSE85215 dataset observations), (2) robust detectability by standard qRT-PCR protocols, and (3) strong correlation with disease pathogenesis. The reliability of our in vitro findings is supported by the use of a well-established HDM-stimulated BEAS-2B cell model, whose inflammatory response characteristics have been amply documented.^22^^,^^23^ This parallel suggests that, similar to the diagnostic utility demonstrated for tRF-Ala-AGC-2-M4 in lupus nephritis,^28^ our identified tsRNAs could be further explored as candidates for minimally invasive asthma diagnostic tests using readily accessible biological samples like serum or saliva. However, extensive validation in larger clinical cohorts and functional studies are needed before any clinical application can be considered.

Our study highlighted the significant regulatory role of tRF-21-Z4NY2V7KE on MYBL1 mRNA expression, as confirmed by a dual-fluorescent reporter gene system and site-directed mutagenesis. This finding underscores the specific interactions between tsRNAs and their target mRNAs, thus contributing to the understanding of post-transcriptional regulatory mechanisms involved in asthma pathogenesis, which aligns with insights from previous studies demonstrating the impact of small RNA fragments on gene expression and inflammatory processes (eg, the suppression of pro-inflammatory factors and modulation of signaling pathways, as illustrated by the work on tRF-3023b).^29^ From a biological perspective, MYBL1 is a transcription factor involved in cell cycle regulation and differentiation, and its dysregulation has been linked to airway epithelial remodeling in asthma. The tRF-21-Z4NY2V7KE-mediated suppression of MYBL1 may therefore contribute to asthma pathogenesis by modulating epithelial repair processes and inflammatory responses. This interaction may intersect with known asthma-related pathways, such as NF-κB signaling and epithelial barrier function, although the precise molecular mechanisms remain to be elucidated. Ultimately, our findings may pave the way for novel therapeutic strategies targeting tsRNAs in the management of asthma-related inflammation.

Several limitations of this study should be acknowledged. First, phenotypic heterogeneity between the discovery (severe asthma) and validation (GSE201955, n = 79, including severe asthma endotype features) cohorts may introduce bias, warranting confirmation in uniformly phenotyped populations. Second, the tRF-21-Z4NY2V7KE/MYBL1 axis was validated only in BEAS-2B cells, necessitating future *in vivo* and primary cell studies. Third, our focus on negatively correlated tsRNA-mRNA pairs may overlook other regulatory mechanisms (eg, epigenetic modifications), highlighting the need for multi-omics investigations. Finally, beyond the validated axis, 3 additional dysregulated tsRNAs (tRF-18-SX73IE03, tRF-22-BZBZOS4Y1, tRF-22-MI7O3B1N4) were identified, whose functional roles warrant future investigation.

Despite these limitations, this study presents the first comprehensive tsRNA-mRNA regulatory network in bronchial epithelial cells from severe asthma patients. Our findings reveal novel post-transcriptional mechanisms that may contribute to asthma pathogenesis and identify candidate tsRNAs and their mRNA targets as potential biomarkers, thereby establishing a foundational resource for future mechanistic and therapeutic studies. Further investigations are warranted to elucidate the precise roles of these tsRNAs and assess their translational relevance.

## Abbreviations

ncRNA: Non-coding RNA; circRNAs: Circular RNAs; lncRNAs: Long non-coding RNAs; miRNAs: MicroRNAs; nt: nucleotides; rRNA: Ribosomal RNA; snoRNA: Small nucleolar RNA; piRNA: Piwi-interacting RNA; tRNA: Transfer RNA; tsRNA: tRNA-derived small RNA; mRNA: Messenger RNA; BEC: Bronchial epithelial cells; HDM: House dust mite; GO: Gene Ontology; KEGG: Kyoto Encyclopedia of Genes and Genomes Pathway; cor: Correlation; qRT-PCR: Quantitative real-time PCR; URT: Untranslated Region; MYBL1: MYB proto-oncogene like 1.

## Authors contributions

**Conceptualization:** Lei Shi and Yubao Cui. **Investigation:** Ying Zhou, Hang Lin, Dongmei Zhou, Xinhui Gong and Ximeng Ma. **Methodology:** Ying Zhou, Hang Lin and Dongmei Zhou. **Data curation:** Xinhui Gong and Ximeng Ma. **Formal analysis:** Lei Shi. Funding **acquisition:** Ying Zhou and Yubao Cui. **Project administration:** Yubao Cui. **Resources:** Ying Zhou and Hang Lin. **Software:** Lei Shi. **Validation:** Dongmei Zhou. **Visualization:** Xinhui Gong. **Writing original draft:** Ying Zhou. **Writing – review & editing:** Yubao Cui. The manuscript was written through contributions of all authors. All authors have given approval to the final version of the manuscript.

## Ethics approval and consent to participate

Not applicable.

## Consent for publication

Authors approved the publication of the work.

## Data availability

All data generated or analyzed during this study are included in this article. The datasets used and/or analyzed during the current study are available from the corresponding author on reasonable request.

## Disclosure of the use of generative AI and AI-assisted technologies in the writing process

Nothing to disclose, the author(s) declare that no generative AI was used in the creation of this manuscript.

## Funding

This study was supported by the Taihu Lake talent plan (Top-Level, no. 2020THRC-GD-7), the Medical Key Strategic Project of Wuxi Health Commission (2025TH-LJ-CYB), the “333 Project” of Jiangsu Province in 2022 (ZUZHIBU 202221001), Jiangsu Maternal and Child Health Research Project (no. F202068), and Jiangsu Provincial Research Project on the Prevention and Control of Schistosomiasis and Parasitic Diseases (No. x202316).

## Competing interest

The authors declare no competing interests.
