# Supplementary material for: Integrated transcriptomic analysis identifies tsRNA–mRNA regulatory axis in asthma pathogenesis
Source: World Allergy Organ J. 2026 Apr 28;19(5):101389. doi: 10.1016/j.waojou.2026.101389 (PMC13141492; doi:10.1016/j.waojou.2026.101389)
Supplement: Multimedia component 1 [file mmc1.docx]

**Supplementary Table 1.** The primers used in amplifying tsRNA from BEAS-2B cells stimulated with HDM extracts.

| **Gene names** | **Primers sequences** | **annealing temperature (℃)** | **Product length (bp)** |
| --- | --- | --- | --- |
| U6 | F:5’GCTTCGGCAGCACATATACTAAAAT3’  R:5’CGCTTCACGAATTTGCGTGTCAT3’ | 60 | 89 |
| tRF-18-SX73IE03 | F:5’ TCCGACGATCGTCTAGTGGTA 3'  R:5’ ACGTGTGCTCTTCCGATCTAG 3’ | 60 | 47 |
| tRF-22-BZBZOS4Y1 | F:5’ TTCTACAGTCCGACGATCAACTT 3'  R:5’ CGATCTGTCAGAGCGGTCAA 3’ | 60 | 46 |
| tRF-22-MI7O3B1N4 | F:5’ TACAGTCCGACGATCCGGCT 3'  R:5’ TCTTCCGATCTCCAACCTTTC 3’ | 60 | 48 |
