# Supplementary material for: Integrated transcriptomic analysis identifies tsRNA–mRNA regulatory axis in asthma pathogenesis
Source: World Allergy Organ J. 2026 Apr 28;19(5):101389. doi: 10.1016/j.waojou.2026.101389 (PMC13141492; doi:10.1016/j.waojou.2026.101389)
Supplement: Multimedia component 2 [file mmc2.docx]

**Supplementary Table 2**. The interaction of tsRNA and mRNA in GSE85214

| **tsRNA** | **tsRNA_style** | **tsRNA_Biotype** | **Gene Symbol** | **Ensembl_Gene_ID** | **mRNA_style** | **mRNA_Biotype** |
| --- | --- | --- | --- | --- | --- | --- |
| tRF-17-QK11M3Q | up | tsRNA | CAPN10 | ENSG00000142330.20 | down | mRNA |
| tRF-19-IRMJ6VE2 | up | tsRNA | NMNAT3 | ENSG00000163864.17 | down | mRNA |
| tRF-20-P42U6R93 | up | tsRNA | FGFR3 | ENSG00000068078.19 | down | mRNA |
| tRF-20-P42U6R93 | up | tsRNA | PGAP3 | ENSG00000161395.14 | down | mRNA |
| tRF-20-R2IP4OQ3 | up | tsRNA | SCAMP5 | ENSG00000198794.12 | down | mRNA |
| tRF-20-R2IP4OQ3 | up | tsRNA | SYNGR1 | ENSG00000100321.15 | down | mRNA |
| tRF-21-P2PSSELQB | up | tsRNA | ADM | ENSG00000148926.10 | down | mRNA |
| tRF-21-P2PSSELQB | up | tsRNA | AIFM2 | ENSG00000042286.15 | down | mRNA |
| tRF-21-P2PSSELQB | up | tsRNA | FOSL2 | ENSG00000075426.12 | down | mRNA |
| tRF-21-P2PSSELQB | up | tsRNA | GALNS | ENSG00000141012.13 | down | mRNA |
| tRF-21-P2PSSELQB | up | tsRNA | GTPBP2 | ENSG00000172432.19 | down | mRNA |
| tRF-21-S79PVOEOE | down | tsRNA | AJAP1 | ENSG00000196581.11 | up | mRNA |
| tRF-21-S79PVOEOE | down | tsRNA | RTTN | ENSG00000176225.14 | up | mRNA |
| tRF-21-S79PVOEOE | down | tsRNA | TNIP3 | ENSG00000050730.16 | up | mRNA |
| tRF-21-VKS4I7LZE | up | tsRNA | CAPN5 | ENSG00000149260.18 | down | mRNA |
| tRF-21-Z4NY2V7KE | down | tsRNA | MYBL1 | ENSG00000185697.16 | up | mRNA |
| tRF-21-Z4NY2V7KE | down | tsRNA | NFAM1 | ENSG00000235568.7 | up | mRNA |
| tRF-22-8XF6RE98N | down | tsRNA | BTK | ENSG00000010671.16 | up | mRNA |
| tRF-22-8XF6RE98N | down | tsRNA | SLC9B2 | ENSG00000164038.16 | up | mRNA |
| tRF-23-2IUIX1Q7DR | up | tsRNA | BMP3 | ENSG00000152785.7 | down | mRNA |
| tRF-23-2IUIX1Q7DR | up | tsRNA | PLEKHH2 | ENSG00000152527.14 | down | mRNA |
| tRF-23-WJ9X0UD304 | down | tsRNA | ANKUB1 | ENSG00000206199.11 | up | mRNA |
| tRF-25-YONONU3IND | up | tsRNA | HLF | ENSG00000108924.14 | down | mRNA |
| tRF-25-YONONU3IND | up | tsRNA | SDC1 | ENSG00000115884.11 | down | mRNA |
| tRF-26-IK9NJ4S2I7D | down | tsRNA | ANXA6 | ENSG00000197043.14 | up | mRNA |
| tRF-26-IK9NJ4S2I7D | down | tsRNA | IQCH | ENSG00000103599.20 | up | mRNA |
| tRF-26-IK9NJ4S2I7D | down | tsRNA | LIPG | ENSG00000101670.12 | up | mRNA |
| tRF-26-IK9NJ4S2I7D | down | tsRNA | PAPPA2 | ENSG00000116183.11 | up | mRNA |
| tRF-26-IK9NJ4S2I7D | down | tsRNA | PRR11 | ENSG00000068489.13 | up | mRNA |
| tRF-26-IK9NJ4S2I7D | down | tsRNA | PTCD2 | ENSG00000049883.15 | up | mRNA |
| tRF-26-IK9NJ4S2I7D | down | tsRNA | STEAP1B | ENSG00000105889.15 | up | mRNA |
| tRF-26-IK9NJ4S2I7D | down | tsRNA | TGM2 | ENSG00000198959.12 | up | mRNA |
| tRF-26-IK9NJ4S2I7D | down | tsRNA | TIMM21 | ENSG00000075336.12 | up | mRNA |
| tRF-27-3JVIJMRPFQL | down | tsRNA | SLC39A8 | ENSG00000138821.13 | up | mRNA |
| tRF-27-HJYJRPFQZDP | up | tsRNA | **DCLK1** | ENSG00000133083.15 | down | mRNA |
| tRF-27-HJYJRPFQZDP | up | tsRNA | **FOXP2** | ENSG00000128573.26 | down | mRNA |
| tRF-27-HJYJRPFQZDP | up | tsRNA | **IGFBP3** | ENSG00000146674.15 | down | mRNA |
| tRF-27-HJYJRPFQZDP | up | tsRNA | L3MBTL1 | ENSG00000185513.17 | down | mRNA |
| tRF-27-HJYJRPFQZDP | up | tsRNA | **LOX** | ENSG00000113083.14 | down | mRNA |
| tRF-27-HJYJRPFQZDP | up | tsRNA | LRAT | ENSG00000121207.12 | down | mRNA |
| tRF-27-HJYJRPFQZDP | up | tsRNA | TRIM29 | ENSG00000137699.17 | down | mRNA |
| tRF-27-U5XBINVDRI2 | down | tsRNA | ARL6 | ENSG00000113966.10 | up | mRNA |
| tRF-27-U5XBINVDRI2 | down | tsRNA | ARL9 | ENSG00000196503.5 | up | mRNA |
| tRF-27-U5XBINVDRI2 | down | tsRNA | CBWD3 | ENSG00000196873.16 | up | mRNA |
| tRF-27-U5XBINVDRI2 | down | tsRNA | CCND2 | ENSG00000118971.9 | up | mRNA |
| tRF-27-U5XBINVDRI2 | down | tsRNA | CYP24A1 | ENSG00000019186.10 | up | mRNA |
| tRF-27-U5XBINVDRI2 | down | tsRNA | ENPP1 | ENSG00000197594.13 | up | mRNA |
| tRF-27-U5XBINVDRI2 | down | tsRNA | LPXN | ENSG00000110031.13 | up | mRNA |
| tRF-27-U5XBINVDRI2 | down | tsRNA | LTBP1 | ENSG00000049323.16 | up | mRNA |
| tRF-27-U5XBINVDRI2 | down | tsRNA | NANP | ENSG00000170191.5 | up | mRNA |
| tRF-27-U5XBINVDRI2 | down | tsRNA | NBPF3 | ENSG00000142794.18 | up | mRNA |
| tRF-27-U5XBINVDRI2 | down | tsRNA | PPP1R3C | ENSG00000119938.9 | up | mRNA |
| tRF-27-U5XBINVDRI2 | down | tsRNA | RHOBTB1 | ENSG00000072422.17 | up | mRNA |
| tRF-27-U5XBINVDRI2 | down | tsRNA | SERP2 | ENSG00000151778.11 | up | mRNA |
| tRF-27-U5XBINVDRI2 | down | tsRNA | SLC39A8 | ENSG00000138821.13 | up | mRNA |
| tRF-27-U5XBINVDRI2 | down | tsRNA | SSR3 | ENSG00000114850.7 | up | mRNA |
| tRF-27-U5XBINVDRI2 | down | tsRNA | UBXN8 | ENSG00000104691.15 | up | mRNA |
| tRF-28-H4SXQ3V2Y7DZ | down | tsRNA | BFSP1 | ENSG00000125864.14 | up | mRNA |
| tRF-28-H4SXQ3V2Y7DZ | down | tsRNA | GLT8D2 | ENSG00000120820.12 | up | mRNA |
| tRF-28-H4SXQ3V2Y7DZ | down | tsRNA | MTRR | ENSG00000124275.15 | up | mRNA |
| tRF-28-H4SXQ3V2Y7DZ | down | tsRNA | PRIM1 | ENSG00000198056.15 | up | mRNA |
| tRF-28-H4SXQ3V2Y7DZ | down | tsRNA | THOC7 | ENSG00000163634.12 | up | mRNA |
| tRF-28-H4SXQ3V2Y7DZ | down | tsRNA | XRRA1 | ENSG00000166435.15 | up | mRNA |
| tRF-28-I3VF4YO9XED2 | down | tsRNA | C7orf25 | ENSG00000136197.12 | up | mRNA |
| tRF-28-I3VF4YO9XED2 | down | tsRNA | COX11 | ENSG00000166260.13 | up | mRNA |
| tRF-28-I3VF4YO9XED2 | down | tsRNA | HEPHL1 | ENSG00000181333.12 | up | mRNA |
| tRF-28-I3VF4YO9XED2 | down | tsRNA | KCNJ15 | ENSG00000157551.19 | up | mRNA |
| tRF-28-I3VF4YO9XED2 | down | tsRNA | METTL21A | ENSG00000144401.14 | up | mRNA |
| tRF-28-I3VF4YO9XED2 | down | tsRNA | MLIP | ENSG00000146147.15 | up | mRNA |
| tRF-28-I3VF4YO9XED2 | down | tsRNA | MRS2 | ENSG00000124532.15 | up | mRNA |
| tRF-28-I3VF4YO9XED2 | down | tsRNA | NSL1 | ENSG00000117697.15 | up | mRNA |
| tRF-28-I3VF4YO9XED2 | down | tsRNA | SPA17 | ENSG00000064199.7 | up | mRNA |
| tRF-28-I3VF4YO9XED2 | down | tsRNA | TUB | ENSG00000166402.9 | up | mRNA |
| tRF-28-MQ18Y3E7QN00 | down | tsRNA | CD96 | ENSG00000153283.13 | up | mRNA |
| tRF-28-MQ18Y3E7QN00 | down | tsRNA | CDH6 | ENSG00000113361.13 | up | mRNA |
| tRF-28-MQ18Y3E7QN00 | down | tsRNA | EYS | ENSG00000188107.15 | up | mRNA |
| tRF-28-MQ18Y3E7QN00 | down | tsRNA | IQCH | ENSG00000103599.20 | up | mRNA |
| tRF-28-MQ18Y3E7QN00 | down | tsRNA | SH3TC2 | ENSG00000169247.14 | up | mRNA |
| tRF-28-MQ18Y3E7QN00 | down | tsRNA | SLC46A3 | ENSG00000139508.15 | up | mRNA |
| tRF-28-WS3V2VR0PSDZ | down | tsRNA | NIPA2 | ENSG00000140157.15 | up | mRNA |
| tRF-29-08F4BDNZ8OIR | down | tsRNA | HVCN1 | ENSG00000122986.14 | up | mRNA |
| tRF-29-08F4BDNZ8OIR | down | tsRNA | PTCD2 | ENSG00000049883.15 | up | mRNA |
| tRF-29-4S14IZJQXEJU | down | tsRNA | ANO1 | ENSG00000131620.17 | up | mRNA |
| tRF-29-4S14IZJQXEJU | down | tsRNA | C7orf25 | ENSG00000136197.12 | up | mRNA |
| tRF-29-4S14IZJQXEJU | down | tsRNA | XG | ENSG00000124343.14 | up | mRNA |
| tRF-29-5DMKYUYRLHIX | down | tsRNA | CDKN2A | ENSG00000147889.17 | up | mRNA |
| tRF-29-5DMKYUYRLHIX | down | tsRNA | EXOSC8 | ENSG00000120699.13 | up | mRNA |
| tRF-29-5DMKYUYRLHIX | down | tsRNA | TAGAP | ENSG00000164691.18 | up | mRNA |
| tRF-29-7EMQ18Y3E7IN | down | tsRNA | CCZ1 | ENSG00000122674.12 | up | mRNA |
| tRF-29-7EMQ18Y3E7IN | down | tsRNA | SUB1 | ENSG00000113387.12 | up | mRNA |
| tRF-29-H4SXQ3V2Y72E | down | tsRNA | BFSP1 | ENSG00000125864.14 | up | mRNA |
| tRF-29-H4SXQ3V2Y72E | down | tsRNA | GLT8D2 | ENSG00000120820.12 | up | mRNA |
| tRF-29-H4SXQ3V2Y72E | down | tsRNA | IL1A | ENSG00000115008.6 | up | mRNA |
| tRF-29-H4SXQ3V2Y72E | down | tsRNA | INVS | ENSG00000119509.13 | up | mRNA |
| tRF-29-H4SXQ3V2Y72E | down | tsRNA | MTRR | ENSG00000124275.15 | up | mRNA |
| tRF-29-H4SXQ3V2Y72E | down | tsRNA | PRIM1 | ENSG00000198056.15 | up | mRNA |
| tRF-29-H4SXQ3V2Y72E | down | tsRNA | SLC35G1 | ENSG00000176273.15 | up | mRNA |
| tRF-29-H4SXQ3V2Y72E | down | tsRNA | THOC7 | ENSG00000163634.12 | up | mRNA |
| tRF-29-H4SXQ3V2Y72E | down | tsRNA | TNFSF4 | ENSG00000117586.11 | up | mRNA |
| tRF-29-H4SXQ3V2Y72E | down | tsRNA | XRRA1 | ENSG00000166435.15 | up | mRNA |
| tRF-29-PSQP4PW3FJFL | down | tsRNA | **CEACAM5** | ENSG00000105388.16 | up | mRNA |
| tRF-29-PSQP4PW3FJFL | down | tsRNA | PRR11 | ENSG00000068489.13 | up | mRNA |
| tRF-30-32VIJMRPFQJD | down | tsRNA | RBM24 | ENSG00000112183.15 | up | mRNA |
| tRF-30-623K7SIR3DR2 | down | tsRNA | ISM2 | ENSG00000100593.18 | up | mRNA |
| tRF-30-PSQP4PW3FJI0 | down | tsRNA | CEACAM5 | ENSG00000105388.16 | up | mRNA |
| tRF-30-PSQP4PW3FJI0 | down | tsRNA | PRR11 | ENSG00000068489.13 | up | mRNA |
| tRF-30-PSQP4PW3FJI0 | down | tsRNA | TNFRSF1B | ENSG00000028137.19 | up | mRNA |
| tRF-30-PSQP4PW3FJI0 | down | tsRNA | TNFSF15 | ENSG00000181634.8 | up | mRNA |
| tRF-30-QKF1R3WE8RO8 | down | tsRNA | ANXA6 | ENSG00000197043.14 | up | mRNA |
| tRF-30-QKF1R3WE8RO8 | down | tsRNA | GRK5 | ENSG00000198873.12 | up | mRNA |
| tRF-30-QKF1R3WE8RO8 | down | tsRNA | RPL22L1 | ENSG00000163584.18 | up | mRNA |
| tRF-31-2YU04DYJIO3ZE | down | tsRNA | AGMAT | ENSG00000116771.6 | up | mRNA |
| tRF-31-2YU04DYJIO3ZE | down | tsRNA | C12orf75 | ENSG00000235162.9 | up | mRNA |
| tRF-31-2YU04DYJIO3ZE | down | tsRNA | CCDC148 | ENSG00000153237.18 | up | mRNA |
| tRF-31-2YU04DYJIO3ZE | down | tsRNA | CDCA7L | ENSG00000164649.20 | up | mRNA |
| tRF-31-2YU04DYJIO3ZE | down | tsRNA | CLMP | ENSG00000166250.12 | up | mRNA |
| tRF-31-2YU04DYJIO3ZE | down | tsRNA | DTWD1 | ENSG00000104047.15 | up | mRNA |
| tRF-31-2YU04DYJIO3ZE | down | tsRNA | INVS | ENSG00000119509.13 | up | mRNA |
| tRF-31-2YU04DYJIO3ZE | down | tsRNA | RCHY1 | ENSG00000163743.13 | up | mRNA |
| tRF-31-2YU04DYJIO3ZE | down | tsRNA | SLC4A4 | ENSG00000080493.17 | up | mRNA |
| tRF-31-2YU04DYJIO3ZE | down | tsRNA | SORBS1 | ENSG00000095637.22 | up | mRNA |
| tRF-31-2YU04DYJIO3ZE | down | tsRNA | SSR3 | ENSG00000114850.7 | up | mRNA |
| tRF-31-2YU04DYJIO3ZE | down | tsRNA | TNFSF4 | ENSG00000117586.11 | up | mRNA |
| tRF-31-2YU04DYJIO3ZE | down | tsRNA | UBXN8 | ENSG00000104691.15 | up | mRNA |
| tRF-31-6XQ6S8V0J8O9E | down | tsRNA | CCND2 | ENSG00000118971.9 | up | mRNA |
| tRF-31-6XQ6S8V0J8O9E | down | tsRNA | MRS2 | ENSG00000124532.15 | up | mRNA |
| tRF-31-6XQ6S8V0J8O9E | down | tsRNA | PDCD5 | ENSG00000105185.12 | up | mRNA |
| tRF-31-6XQ6S8V0J8O9E | down | tsRNA | SLC35G1 | ENSG00000176273.15 | up | mRNA |
| tRF-31-6XQ6S8V0J8O9E | down | tsRNA | ZNF280B | ENSG00000275004.4 | up | mRNA |
| tRF-31-FSXMSL73VL4YD | down | tsRNA | ANKDD1A | ENSG00000166839.17 | up | mRNA |
| tRF-31-FSXMSL73VL4YD | down | tsRNA | COL13A1 | ENSG00000197467.15 | up | mRNA |
| tRF-31-FSXMSL73VL4YD | down | tsRNA | DONSON | ENSG00000159147.18 | up | mRNA |
| tRF-31-FSXMSL73VL4YD | down | tsRNA | FMOD | ENSG00000122176.12 | up | mRNA |
| tRF-31-K84J83ML5FX2D | down | tsRNA | MYOZ3 | ENSG00000164591.14 | up | mRNA |
| tRF-31-K84J83ML5FX2D | down | tsRNA | PRR11 | ENSG00000068489.13 | up | mRNA |
| tRF-31-M2OSRNLNKSEK0 | down | tsRNA | ANXA6 | ENSG00000197043.14 | up | mRNA |
| tRF-31-M2OSRNLNKSEK0 | down | tsRNA | ARL9 | ENSG00000196503.5 | up | mRNA |
| tRF-31-M2OSRNLNKSEK0 | down | tsRNA | CDKN2A | ENSG00000147889.17 | up | mRNA |
| tRF-31-M2OSRNLNKSEK0 | down | tsRNA | GCSH | ENSG00000140905.11 | up | mRNA |
| tRF-31-M2OSRNLNKSEK0 | down | tsRNA | IL7R | ENSG00000168685.15 | up | mRNA |
| tRF-31-M2OSRNLNKSEK0 | down | tsRNA | KCNQ5 | ENSG00000185760.16 | up | mRNA |
| tRF-31-M2OSRNLNKSEK0 | down | tsRNA | NFAM1 | ENSG00000235568.7 | up | mRNA |
| tRF-31-M2OSRNLNKSEK0 | down | tsRNA | NFU1 | ENSG00000169599.13 | up | mRNA |
| tRF-31-M2OSRNLNKSEK0 | down | tsRNA | SLC6A11 | ENSG00000132164.10 | up | mRNA |
| tRF-31-M2OSRNLNKSEK0 | down | tsRNA | ST6GALNAC5 | ENSG00000117069.15 | up | mRNA |
| tRF-31-M2OSRNLNKSEK0 | down | tsRNA | TMEM97 | ENSG00000109084.14 | up | mRNA |
| tRF-31-PIR8YP9LON4VD | down | tsRNA | AGPAT5 | ENSG00000155189.12 | up | mRNA |
| tRF-31-PIR8YP9LON4VD | down | tsRNA | AJAP1 | ENSG00000196581.11 | up | mRNA |
| tRF-31-PIR8YP9LON4VD | down | tsRNA | CD47 | ENSG00000196776.16 | up | mRNA |
| tRF-31-PIR8YP9LON4VD | down | tsRNA | CDKN3 | ENSG00000100526.20 | up | mRNA |
| tRF-31-PIR8YP9LON4VD | down | tsRNA | CHPT1 | ENSG00000111666.11 | up | mRNA |
| tRF-31-PIR8YP9LON4VD | down | tsRNA | CSF3 | ENSG00000108342.13 | up | mRNA |
| tRF-31-PIR8YP9LON4VD | down | tsRNA | DNAH2 | ENSG00000183914.14 | up | mRNA |
| tRF-31-PIR8YP9LON4VD | down | tsRNA | ENPP1 | ENSG00000197594.13 | up | mRNA |
| tRF-31-PIR8YP9LON4VD | down | tsRNA | ERC2 | ENSG00000187672.14 | up | mRNA |
| tRF-31-PIR8YP9LON4VD | down | tsRNA | IL7R | ENSG00000168685.15 | up | mRNA |
| tRF-31-PIR8YP9LON4VD | down | tsRNA | KIF6 | ENSG00000164627.18 | up | mRNA |
| tRF-31-PIR8YP9LON4VD | down | tsRNA | LIPG | ENSG00000101670.12 | up | mRNA |
| tRF-31-PIR8YP9LON4VD | down | tsRNA | LMO3 | ENSG00000048540.15 | up | mRNA |
| tRF-31-PIR8YP9LON4VD | down | tsRNA | LRRC34 | ENSG00000171757.17 | up | mRNA |
| tRF-31-PIR8YP9LON4VD | down | tsRNA | MYO1F | ENSG00000142347.19 | up | mRNA |
| tRF-31-PIR8YP9LON4VD | down | tsRNA | PHTF1 | ENSG00000116793.16 | up | mRNA |
| tRF-31-PIR8YP9LON4VD | down | tsRNA | PLA2G4C | ENSG00000105499.14 | up | mRNA |
| tRF-31-PIR8YP9LON4VD | down | tsRNA | PRR11 | ENSG00000068489.13 | up | mRNA |
| tRF-31-PIR8YP9LON4VD | down | tsRNA | PTCD2 | ENSG00000049883.15 | up | mRNA |
| tRF-31-PIR8YP9LON4VD | down | tsRNA | RTTN | ENSG00000176225.14 | up | mRNA |
| tRF-31-PIR8YP9LON4VD | down | tsRNA | TBC1D1 | ENSG00000065882.16 | up | mRNA |
| tRF-31-PIR8YP9LON4VD | down | tsRNA | TCP1 | ENSG00000120438.12 | up | mRNA |
| tRF-31-PIR8YP9LON4VD | down | tsRNA | TNFSF15 | ENSG00000181634.8 | up | mRNA |
| tRF-31-PIR8YP9LON4VD | down | tsRNA | ZPLD1 | ENSG00000170044.8 | up | mRNA |
| tRF-32-389MV47P596V5 | down | tsRNA | ABCC4 | ENSG00000125257.16 | up | mRNA |
| tRF-32-389MV47P596V5 | down | tsRNA | EPM2A | ENSG00000112425.16 | up | mRNA |
| tRF-32-389MV47P596V5 | down | tsRNA | FMOD | ENSG00000122176.12 | up | mRNA |
| tRF-32-389MV47P596V5 | down | tsRNA | GOLGA7B | ENSG00000155265.11 | up | mRNA |
| tRF-32-389MV47P596V5 | down | tsRNA | IGF2BP3 | ENSG00000136231.14 | up | mRNA |
| tRF-32-389MV47P596V5 | down | tsRNA | KIF6 | ENSG00000164627.18 | up | mRNA |
| tRF-32-389MV47P596V5 | down | tsRNA | LIPG | ENSG00000101670.12 | up | mRNA |
| tRF-32-389MV47P596V5 | down | tsRNA | MYOZ3 | ENSG00000164591.14 | up | mRNA |
| tRF-32-389MV47P596V5 | down | tsRNA | OAS2 | ENSG00000111335.13 | up | mRNA |
| tRF-32-389MV47P596V5 | down | tsRNA | PDPN | ENSG00000162493.16 | up | mRNA |
| tRF-32-389MV47P596V5 | down | tsRNA | PNMA2 | ENSG00000240694.9 | up | mRNA |
| tRF-32-389MV47P596V5 | down | tsRNA | RNF8 | ENSG00000112130.17 | up | mRNA |
| tRF-32-389MV47P596V5 | down | tsRNA | RWDD2B | ENSG00000156253.7 | up | mRNA |
| tRF-32-389MV47P596V5 | down | tsRNA | SH2D4B | ENSG00000178217.14 | up | mRNA |
| tRF-32-389MV47P596V5 | down | tsRNA | SH3TC2 | ENSG00000169247.14 | up | mRNA |
| tRF-32-389MV47P596V5 | down | tsRNA | SKP2 | ENSG00000145604.16 | up | mRNA |
| tRF-32-389MV47P596V5 | down | tsRNA | ST6GALNAC5 | ENSG00000117069.15 | up | mRNA |
| tRF-32-389MV47P596V5 | down | tsRNA | STAMBPL1 | ENSG00000138134.12 | up | mRNA |
| tRF-32-389MV47P596V5 | down | tsRNA | TBC1D1 | ENSG00000065882.16 | up | mRNA |
| tRF-32-389MV47P596V5 | down | tsRNA | TPM1 | ENSG00000140416.23 | up | mRNA |
| tRF-32-389MV47P596V5 | down | tsRNA | TRDMT1 | ENSG00000107614.22 | up | mRNA |
| tRF-32-389MV47P596V5 | down | tsRNA | ZBED2 | ENSG00000177494.6 | up | mRNA |
| tRF-32-389MV47P596V5 | down | tsRNA | ZPLD1 | ENSG00000170044.8 | up | mRNA |
| tRF-32-M1M3WD8S746D2 | down | tsRNA | TUBB3 | ENSG00000258947.7 | up | mRNA |
| tRF-32-MIF91SS2P46I3 | down | tsRNA | BPGM | ENSG00000172331.12 | up | mRNA |
| tRF-32-MIF91SS2P46I3 | down | tsRNA | EPM2A | ENSG00000112425.16 | up | mRNA |
| tRF-32-MIF91SS2P46I3 | down | tsRNA | MMP19 | ENSG00000123342.16 | up | mRNA |
| tRF-32-MIF91SS2P46I3 | down | tsRNA | TRAF1 | ENSG00000056558.11 | up | mRNA |
| tRF-38-HMI8W47W1R7HFEV | up | tsRNA | ARHGAP6 | ENSG00000047648.23 | down | mRNA |
| tRF-38-HMI8W47W1R7HFEV | up | tsRNA | ARMC5 | ENSG00000140691.18 | down | mRNA |
| tRF-38-HMI8W47W1R7HFEV | up | tsRNA | BMP3 | ENSG00000152785.7 | down | mRNA |
| tRF-38-HMI8W47W1R7HFEV | up | tsRNA | C1RL | ENSG00000139178.11 | down | mRNA |
| tRF-38-HMI8W47W1R7HFEV | up | tsRNA | CYP3A5 | ENSG00000106258.15 | down | mRNA |
| tRF-38-HMI8W47W1R7HFEV | up | tsRNA | EGR3 | ENSG00000179388.9 | down | mRNA |
| tRF-38-HMI8W47W1R7HFEV | up | tsRNA | EVPLL | ENSG00000214860.5 | down | mRNA |
| tRF-38-HMI8W47W1R7HFEV | up | tsRNA | FOXQ1 | ENSG00000164379.7 | down | mRNA |
| tRF-38-HMI8W47W1R7HFEV | up | tsRNA | GJB2 | ENSG00000165474.8 | down | mRNA |
| tRF-38-HMI8W47W1R7HFEV | up | tsRNA | HLF | ENSG00000108924.14 | down | mRNA |
| tRF-38-HMI8W47W1R7HFEV | up | tsRNA | LFNG | ENSG00000106003.13 | down | mRNA |
| tRF-38-HMI8W47W1R7HFEV | up | tsRNA | LNX1 | ENSG00000072201.14 | down | mRNA |
| tRF-38-HMI8W47W1R7HFEV | up | tsRNA | LRAT | ENSG00000121207.12 | down | mRNA |
| tRF-38-HMI8W47W1R7HFEV | up | tsRNA | LRRC4 | ENSG00000128594.8 | down | mRNA |
| tRF-38-HMI8W47W1R7HFEV | up | tsRNA | NIT1 | ENSG00000158793.14 | down | mRNA |
| tRF-38-HMI8W47W1R7HFEV | up | tsRNA | NKX2-1 | ENSG00000136352.18 | down | mRNA |
| tRF-38-HMI8W47W1R7HFEV | up | tsRNA | PCDHA5 | ENSG00000204965.9 | down | mRNA |
| tRF-38-HMI8W47W1R7HFEV | up | tsRNA | PGAP3 | ENSG00000161395.14 | down | mRNA |
| tRF-38-HMI8W47W1R7HFEV | up | tsRNA | PIWIL2 | ENSG00000197181.12 | down | mRNA |
| tRF-38-HMI8W47W1R7HFEV | up | tsRNA | PLEKHH2 | ENSG00000152527.14 | down | mRNA |
| tRF-38-HMI8W47W1R7HFEV | up | tsRNA | PPP1R9A | ENSG00000158528.12 | down | mRNA |
| tRF-38-HMI8W47W1R7HFEV | up | tsRNA | RBM11 | ENSG00000185272.14 | down | mRNA |
| tRF-38-HMI8W47W1R7HFEV | up | tsRNA | RDH10 | ENSG00000121039.10 | down | mRNA |
| tRF-38-HMI8W47W1R7HFEV | up | tsRNA | SERPINB13 | ENSG00000197641.12 | down | mRNA |
| tRF-38-HMI8W47W1R7HFEV | up | tsRNA | SLC38A10 | ENSG00000157637.13 | down | mRNA |
| tRF-38-HMI8W47W1R7HFEV | up | tsRNA | TMEM47 | ENSG00000147027.4 | down | mRNA |
| tRF-38-HMI8W47W1R7HFEV | up | tsRNA | TSPAN11 | ENSG00000110900.16 | down | mRNA |
| tRF-40-B9I1KQSX0DIJZ726 | up | tsRNA | C10orf99 | ENSG00000188373.5 | down | mRNA |
| tRF-40-B9I1KQSX0DIJZ726 | up | tsRNA | CYP1B1 | ENSG00000138061.12 | down | mRNA |
| tRF-40-B9I1KQSX0DIJZ726 | up | tsRNA | FAM131B | ENSG00000159784.18 | down | mRNA |
| tRF-40-B9I1KQSX0DIJZ726 | up | tsRNA | LRRC4 | ENSG00000128594.8 | down | mRNA |
| tRF-40-B9I1KQSX0DIJZ726 | up | tsRNA | LSM14B | ENSG00000149657.20 | down | mRNA |
| tRF-40-B9I1KQSX0DIJZ726 | up | tsRNA | MFGE8 | ENSG00000140545.15 | down | mRNA |
| tRF-40-B9I1KQSX0DIJZ726 | up | tsRNA | RDH12 | ENSG00000139988.10 | down | mRNA |
| tRF-40-B9I1KQSX0DIJZ726 | up | tsRNA | SV2B | ENSG00000185518.11 | down | mRNA |
| tRF-40-B9I1KQSX0DIJZ726 | up | tsRNA | VTCN1 | ENSG00000134258.17 | down | mRNA |
| tRF-40-VEX0K3XN13QXQWUI | up | tsRNA | C1R | ENSG00000159403.18 | down | mRNA |
| tRF-40-VEX0K3XN13QXQWUI | up | tsRNA | EPGN | ENSG00000182585.10 | down | mRNA |
| tRF-40-VEX0K3XN13QXQWUI | up | tsRNA | EPHA4 | ENSG00000116106.12 | down | mRNA |
| tRF-40-VEX0K3XN13QXQWUI | up | tsRNA | GPR37 | ENSG00000170775.3 | down | mRNA |
| tRF-40-VEX0K3XN13QXQWUI | up | tsRNA | GULP1 | ENSG00000144366.16 | down | mRNA |
| tRF-40-VEX0K3XN13QXQWUI | up | tsRNA | MILR1 | ENSG00000271605.6 | down | mRNA |
| tRF-40-VEX0K3XN13QXQWUI | up | tsRNA | PCDHA5 | ENSG00000204965.9 | down | mRNA |
| tRF-40-VEX0K3XN13QXQWUI | up | tsRNA | RBM20 | ENSG00000203867.8 | down | mRNA |
| tRF-40-VEX0K3XN13QXQWUI | up | tsRNA | TTC39A | ENSG00000085831.15 | down | mRNA |
| tRF-41-8L8NRS9NS334L2H1B | up | tsRNA | ADM | ENSG00000148926.10 | down | mRNA |
| tRF-41-8L8NRS9NS334L2H1B | up | tsRNA | BLNK | ENSG00000095585.17 | down | mRNA |
| tRF-41-8L8NRS9NS334L2H1B | up | tsRNA | C11orf54 | ENSG00000182919.15 | down | mRNA |
| tRF-41-8L8NRS9NS334L2H1B | up | tsRNA | C1RL | ENSG00000139178.11 | down | mRNA |
| tRF-41-8L8NRS9NS334L2H1B | up | tsRNA | C2CD4A | ENSG00000198535.5 | down | mRNA |
| tRF-41-8L8NRS9NS334L2H1B | up | tsRNA | CALCOCO1 | ENSG00000012822.16 | down | mRNA |
| tRF-41-8L8NRS9NS334L2H1B | up | tsRNA | CCDC97 | ENSG00000142039.4 | down | mRNA |
| tRF-41-8L8NRS9NS334L2H1B | up | tsRNA | DEPTOR | ENSG00000155792.10 | down | mRNA |
| tRF-41-8L8NRS9NS334L2H1B | up | tsRNA | EVPLL | ENSG00000214860.5 | down | mRNA |
| tRF-41-8L8NRS9NS334L2H1B | up | tsRNA | FOXP2 | ENSG00000128573.26 | down | mRNA |
| tRF-41-8L8NRS9NS334L2H1B | up | tsRNA | FOXQ1 | ENSG00000164379.7 | down | mRNA |
| tRF-41-8L8NRS9NS334L2H1B | up | tsRNA | GJB2 | ENSG00000165474.8 | down | mRNA |
| tRF-41-8L8NRS9NS334L2H1B | up | tsRNA | GTPBP2 | ENSG00000172432.19 | down | mRNA |
| tRF-41-8L8NRS9NS334L2H1B | up | tsRNA | IRF1 | ENSG00000125347.14 | down | mRNA |
| tRF-41-8L8NRS9NS334L2H1B | up | tsRNA | KLK10 | ENSG00000129451.12 | down | mRNA |
| tRF-41-8L8NRS9NS334L2H1B | up | tsRNA | KRT80 | ENSG00000167767.14 | down | mRNA |
| tRF-41-8L8NRS9NS334L2H1B | up | tsRNA | RNF185 | ENSG00000138942.16 | down | mRNA |
| tRF-41-8L8NRS9NS334L2H1B | up | tsRNA | SCN9A | ENSG00000169432.18 | down | mRNA |
| tRF-41-8L8NRS9NS334L2H1B | up | tsRNA | SLC25A27 | ENSG00000153291.16 | down | mRNA |
| tRF-41-8L8NRS9NS334L2H1B | up | tsRNA | TCEA3 | ENSG00000204219.11 | down | mRNA |
| tRF-41-8L8NRS9NS334L2H1B | up | tsRNA | TP73 | ENSG00000078900.15 | down | mRNA |
| tRF-41-U5YKFN8DYDZDL9X1B | up | tsRNA | BMP3 | ENSG00000152785.7 | down | mRNA |
| tRF-41-U5YKFN8DYDZDL9X1B | up | tsRNA | CATSPER2 | ENSG00000166762.19 | down | mRNA |
| tRF-41-U5YKFN8DYDZDL9X1B | up | tsRNA | HLF | ENSG00000108924.14 | down | mRNA |
| tRF-41-U5YKFN8DYDZDL9X1B | up | tsRNA | ID4 | ENSG00000172201.12 | down | mRNA |
| tRF-41-U5YKFN8DYDZDL9X1B | up | tsRNA | NEBL | ENSG00000078114.19 | down | mRNA |
| tRF-41-U5YKFN8DYDZDL9X1B | up | tsRNA | NMNAT3 | ENSG00000163864.17 | down | mRNA |
| tRF-41-U5YKFN8DYDZDL9X1B | up | tsRNA | PCDHA5 | ENSG00000204965.9 | down | mRNA |
| tRF-41-U5YKFN8DYDZDL9X1B | up | tsRNA | PLEKHH2 | ENSG00000152527.14 | down | mRNA |
| tRF-41-U5YKFN8DYDZDL9X1B | up | tsRNA | PPARGC1A | ENSG00000109819.9 | down | mRNA |
| tRF-41-U5YKFN8DYDZDL9X1B | up | tsRNA | RDH10 | ENSG00000121039.10 | down | mRNA |
| tRF-41-U5YKFN8DYDZDL9X1B | up | tsRNA | SLC10A6 | ENSG00000145283.8 | down | mRNA |
| tRF-41-XENDBP1IUUK7VZ0RB | up | tsRNA | AATK | ENSG00000181409.14 | down | mRNA |
| tRF-41-XENDBP1IUUK7VZ0RB | up | tsRNA | ASAP3 | ENSG00000088280.19 | down | mRNA |
| tRF-41-XENDBP1IUUK7VZ0RB | up | tsRNA | AZGP1 | ENSG00000160862.13 | down | mRNA |
| tRF-41-XENDBP1IUUK7VZ0RB | up | tsRNA | C11orf54 | ENSG00000182919.15 | down | mRNA |
| tRF-41-XENDBP1IUUK7VZ0RB | up | tsRNA | CACNA2D4 | ENSG00000151062.15 | down | mRNA |
| tRF-41-XENDBP1IUUK7VZ0RB | up | tsRNA | CALCOCO1 | ENSG00000012822.16 | down | mRNA |
| tRF-41-XENDBP1IUUK7VZ0RB | up | tsRNA | CAMK1D | ENSG00000183049.13 | down | mRNA |
| tRF-41-XENDBP1IUUK7VZ0RB | up | tsRNA | DCLK1 | ENSG00000133083.15 | down | mRNA |
| tRF-41-XENDBP1IUUK7VZ0RB | up | tsRNA | FLVCR2 | ENSG00000119686.10 | down | mRNA |
| tRF-41-XENDBP1IUUK7VZ0RB | up | tsRNA | FOXP2 | ENSG00000128573.26 | down | mRNA |
| tRF-41-XENDBP1IUUK7VZ0RB | up | tsRNA | GULP1 | ENSG00000144366.16 | down | mRNA |
| tRF-41-XENDBP1IUUK7VZ0RB | up | tsRNA | NIT1 | ENSG00000158793.14 | down | mRNA |
| tRF-41-XENDBP1IUUK7VZ0RB | up | tsRNA | NMNAT3 | ENSG00000163864.17 | down | mRNA |
| tRF-41-XENDBP1IUUK7VZ0RB | up | tsRNA | NOTCH3 | ENSG00000074181.9 | down | mRNA |
| tRF-41-XENDBP1IUUK7VZ0RB | up | tsRNA | OCLN | ENSG00000197822.11 | down | mRNA |
| tRF-41-XENDBP1IUUK7VZ0RB | up | tsRNA | PLEKHH2 | ENSG00000152527.14 | down | mRNA |
| tRF-41-XENDBP1IUUK7VZ0RB | up | tsRNA | SCAMP5 | ENSG00000198794.12 | down | mRNA |
| tRF-41-XENDBP1IUUK7VZ0RB | up | tsRNA | SCN3B | ENSG00000166257.9 | down | mRNA |
| tRF-41-XENDBP1IUUK7VZ0RB | up | tsRNA | SLC35E4 | ENSG00000100036.13 | down | mRNA |
| tRF-41-XENDBP1IUUK7VZ0RB | up | tsRNA | SLC40A1 | ENSG00000138449.11 | down | mRNA |
| tRF-41-XENDBP1IUUK7VZ0RB | up | tsRNA | TREM1 | ENSG00000124731.13 | down | mRNA |
| tRF-41-XENDBP1IUUK7VZ0RB | up | tsRNA | ZNF66 | ENSG00000160229.13 | down | mRNA |
| tRF-45-BZ0IV25Z2IUIX1Q7O6 | up | tsRNA | ARHGAP6 | ENSG00000047648.23 | down | mRNA |
| tRF-45-BZ0IV25Z2IUIX1Q7O6 | up | tsRNA | BLNK | ENSG00000095585.17 | down | mRNA |
| tRF-45-BZ0IV25Z2IUIX1Q7O6 | up | tsRNA | C11orf54 | ENSG00000182919.15 | down | mRNA |
| tRF-45-BZ0IV25Z2IUIX1Q7O6 | up | tsRNA | CIITA | ENSG00000179583.19 | down | mRNA |
| tRF-45-BZ0IV25Z2IUIX1Q7O6 | up | tsRNA | COL21A1 | ENSG00000124749.17 | down | mRNA |
| tRF-45-BZ0IV25Z2IUIX1Q7O6 | up | tsRNA | CYP3A5 | ENSG00000106258.15 | down | mRNA |
| tRF-45-BZ0IV25Z2IUIX1Q7O6 | up | tsRNA | DLX5 | ENSG00000105880.7 | down | mRNA |
| tRF-45-BZ0IV25Z2IUIX1Q7O6 | up | tsRNA | ELF5 | ENSG00000135374.11 | down | mRNA |
| tRF-45-BZ0IV25Z2IUIX1Q7O6 | up | tsRNA | LRRC4 | ENSG00000128594.8 | down | mRNA |
| tRF-45-BZ0IV25Z2IUIX1Q7O6 | up | tsRNA | MMP28 | ENSG00000271447.6 | down | mRNA |
| tRF-45-BZ0IV25Z2IUIX1Q7O6 | up | tsRNA | NEBL | ENSG00000078114.19 | down | mRNA |
| tRF-45-BZ0IV25Z2IUIX1Q7O6 | up | tsRNA | PER3 | ENSG00000049246.14 | down | mRNA |
| tRF-45-BZ0IV25Z2IUIX1Q7O6 | up | tsRNA | PPP1R9A | ENSG00000158528.12 | down | mRNA |
| tRF-45-BZ0IV25Z2IUIX1Q7O6 | up | tsRNA | SPTSSB | ENSG00000196542.8 | down | mRNA |
| tRF-45-BZ0IV25Z2IUIX1Q7O6 | up | tsRNA | TMPRSS4 | ENSG00000137648.19 | down | mRNA |
| tRF-45-BZ0IV25Z2IUIX1Q7O6 | up | tsRNA | TOX2 | ENSG00000124191.18 | down | mRNA |
| tRF-45-BZ0IV25Z2IUIX1Q7O6 | up | tsRNA | TOX3 | ENSG00000103460.17 | down | mRNA |
| tRF-45-BZ0IV25Z2IUIX1Q7O6 | up | tsRNA | ZNF66 | ENSG00000160229.13 | down | mRNA |
| tRF-47-5BF900BY4D84KRIMUF1 | up | tsRNA | ATOH8 | ENSG00000168874.13 | down | mRNA |
| tRF-47-5BF900BY4D84KRIMUF1 | up | tsRNA | CATSPER2 | ENSG00000166762.19 | down | mRNA |
| tRF-47-5BF900BY4D84KRIMUF1 | up | tsRNA | CD36 | ENSG00000135218.19 | down | mRNA |
| tRF-47-5BF900BY4D84KRIMUF1 | up | tsRNA | CERS3 | ENSG00000154227.13 | down | mRNA |
| tRF-47-5BF900BY4D84KRIMUF1 | up | tsRNA | CRABP2 | ENSG00000143320.9 | down | mRNA |
| tRF-47-5BF900BY4D84KRIMUF1 | up | tsRNA | DAPL1 | ENSG00000163331.12 | down | mRNA |
| tRF-47-5BF900BY4D84KRIMUF1 | up | tsRNA | DCLK1 | ENSG00000133083.15 | down | mRNA |
| tRF-47-5BF900BY4D84KRIMUF1 | up | tsRNA | DEPTOR | ENSG00000155792.10 | down | mRNA |
| tRF-47-5BF900BY4D84KRIMUF1 | up | tsRNA | ETS2 | ENSG00000157557.13 | down | mRNA |
| tRF-47-5BF900BY4D84KRIMUF1 | up | tsRNA | FIBIN | ENSG00000176971.4 | down | mRNA |
| tRF-47-5BF900BY4D84KRIMUF1 | up | tsRNA | FOXP2 | ENSG00000128573.26 | down | mRNA |
| tRF-47-5BF900BY4D84KRIMUF1 | up | tsRNA | GLUL | ENSG00000135821.19 | down | mRNA |
| tRF-47-5BF900BY4D84KRIMUF1 | up | tsRNA | GNAO1 | ENSG00000087258.16 | down | mRNA |
| tRF-47-5BF900BY4D84KRIMUF1 | up | tsRNA | HPSE | ENSG00000173083.15 | down | mRNA |
| tRF-47-5BF900BY4D84KRIMUF1 | up | tsRNA | IFFO2 | ENSG00000169991.11 | down | mRNA |
| tRF-47-5BF900BY4D84KRIMUF1 | up | tsRNA | IFITM10 | ENSG00000244242.2 | down | mRNA |
| tRF-47-5BF900BY4D84KRIMUF1 | up | tsRNA | KIFC2 | ENSG00000167702.13 | down | mRNA |
| tRF-47-5BF900BY4D84KRIMUF1 | up | tsRNA | NOTCH3 | ENSG00000074181.9 | down | mRNA |
| tRF-47-5BF900BY4D84KRIMUF1 | up | tsRNA | OLFML2A | ENSG00000185585.20 | down | mRNA |
| tRF-47-5BF900BY4D84KRIMUF1 | up | tsRNA | PCDHA5 | ENSG00000204965.9 | down | mRNA |
| tRF-47-5BF900BY4D84KRIMUF1 | up | tsRNA | PPARGC1A | ENSG00000109819.9 | down | mRNA |
| tRF-47-5BF900BY4D84KRIMUF1 | up | tsRNA | PPP1R9A | ENSG00000158528.12 | down | mRNA |
| tRF-47-5BF900BY4D84KRIMUF1 | up | tsRNA | RALGPS1 | ENSG00000136828.19 | down | mRNA |
| tRF-47-5BF900BY4D84KRIMUF1 | up | tsRNA | RBM4 | ENSG00000173933.21 | down | mRNA |
| tRF-47-5BF900BY4D84KRIMUF1 | up | tsRNA | RNASET2 | ENSG00000026297.16 | down | mRNA |
| tRF-47-5BF900BY4D84KRIMUF1 | up | tsRNA | RNF39 | ENSG00000204618.8 | down | mRNA |
| tRF-47-5BF900BY4D84KRIMUF1 | up | tsRNA | TMEM140 | ENSG00000146859.6 | down | mRNA |
| tRF-47-5BF900BY4D84KRIMUF1 | up | tsRNA | TTC39A | ENSG00000085831.15 | down | mRNA |
